# CNN-BiLSTM-Based Hybrid Deep Learning for Multi-Metric Anomaly Detection and Mitigation in Secure IoMT Healthcare WBANs

**DOI:** 10.3390/s26123849

**Published:** 2026-06-17

**Authors:** Shanmugaraj Muthupandian, Devendran Manoj Kumar

**Affiliations:** Department of Electronics and Communication Engineering, Faculty of Engineering and Technology, SRM Institute of Science and Technology, Ramapuram Campus, Chennai 600089, Tamil Nadu, India

**Keywords:** IoMT, continuous health monitoring, privacy breaches, NodeMCU, CNN + BiLSTM anomaly detection, cybersecurity

## Abstract

Wireless Body Area Networks (WBANs) have become an essential component of modern Internet of Medical Things (IoMT) healthcare systems, enabling continuous monitoring of patient physiological signals through wearable sensors. Despite their advantages, WBAN environments remain highly prone to cyber threats, privacy breaches, and single points of failure. To address these risks, this work proposes a Hybrid Multi-Metric Anomaly Detection (HM-MAD) framework deployed on the NodeMCU-32S platform with BLE 5.0 connectivity for secure continuous glucose monitoring (CGM) data transmission. The detection model simultaneously analyses physiological signals, system-level parameters, and network-level communication metrics, enabling the reliable identification of multiple cyberattacks. The proposed system focuses on securing data transmission against relay attacks, where attackers induce communication delay without modifying payloads, potentially leading to false glucose readings, improper insulin dosage delivery, unauthorized control or denial-of-service. The Convolutional Neural Network (CNN) and Bi-Directional Long Short Term Memory (BiLSTM) model classifies attack types including timing manipulation, replay attacks, power glitches, firmware tampering, and sensor spoofing. Experimental evaluation demonstrates that the proposed CNN + BiLSTM framework achieves 94.6% detection accuracy with an average inference latency of 15 ms, representing a 50% latency reduction compared to Transformer-based intrusion detection models (30 ms), while simultaneously reducing computational overhead by 28% in terms of floating-point operations and memory utilization. These results indicate that the HM-MAD framework provides an effective and scalable solution for protecting resource-constrained IoMT healthcare systems against emerging cyber threats.

## 1. Introduction

The proposed study for securing Wireless Body Networks (WBANs) in the Internet of Medical Things (IoMT) [1] integrates a Convolutional Neural Network (CNN) and Bi-Directional Long Short Term Memory (BiLSTM) [2] with a Hybrid Multi-Metric Anomaly Detection and Mitigation (HM-MAD) framework for securing WBANs in IoMT. In contrast to prior studies that rely on cloud or server-based processing, this work demonstrates a fully functional on-device threat detection and mitigation system deployed on NodeMCU-32S, enabling the real-time low-latency protection in IoMT/WBANs. The process begins when an attacker eavesdrops by placing a network interface card (NIC) into promiscuous mode or using specialized sniffing hardware such as Wi-Fi adapters with monitor mode, Zigbee sniffers, or Software Defined Radios (SDRs) to capture all packets traversing wireless devices like wearable continuous glucose monitoring (CGM) sensors [3] or through a wired medium. Once in this mode, the attacker passively listens to ongoing communications, collecting raw packets without alerting the sender or receiver. These packets contain headers with metadata such as source and destination IP addresses, enabling the attacker to gradually build a profile of the network’s structure and vulnerabilities. This initial step of eavesdropping leads to advanced attacks such as session hijacking, replay, or man-in-the-middle (MITM) attack.

The data packets of the form [Packet 0: Glucose = 110, Temp = 36.2, 1] are sniffed by intercepting sensor-to-gateway traffic and induce an abnormal timing pattern. In WBANs, medical sensors like glucose monitors and temperature patches generate small packets of readings at regular intervals, typically every 50 microseconds with a tolerance of ±5 microseconds. A relay attacker induces an additional delay of around 200 microseconds, creating timing irregularities that can be flagged as suspicious by the threat detection system monitoring this traffic. A legitimate sender maintains consistent timing with minimal jitter or variance, while a relay attacker causes small, random jitters due to buffering and retransmission delays. The 200-microsecond induced delay is a unifying footprint across all major relay attack types in WBANs like sensor spoofing, power-line relaying, timing exploitation, resets, or data injection. Intrusion detection and machine learning-based systems use microsecond-level timing analysis as a universal and effective method to identify such relay attacks. The IoMT data logs used in the proposed system include sensor physiological readings (glucose, sensor noise, and temperature), network parameters (Received Signal Strength Indicator (RSSI), Signal-to-Noise Ratio (SNR), packet delay, jitter, packet loss, duplicate rate, replay flag, Wi-Fi Basic Service Set Identifier (BSSID), channel, transmission power (Tx) power, and authentication) and system parameters (pin event, reset count, boot reason, voltage, current, Analog-to-Digital Converter (ADC) value, Serial Peripheral Interface (SPI) errors, Universal Asynchronous Receiver-Transmitter (UART) overruns, secure boot, and duty cycle). These entries serve as a baseline for inducing cyberattack scenarios. The CNN and BiLSTM model classify attacks like timing manipulation [4], firmware tampering [5], power glitch [6], sensor spoofing [7] and replay attacks [8] in real time. The study presents a Hybrid Multi-Metric Anomaly Detection and Mitigation system (HM-MAD), an AI deep learning model trained to recognize timing irregularities and anomalous variations. The proposed system effectively detects and flags potential relay attacks in real-time, providing threat detection, privacy-aware security to WBAN-based healthcare systems, and a solid framework for healthcare providers and patients.

### 1.1. Research Motivation

As more WBANs are incorporated into healthcare IoMT systems, critical security and performance issues arise in real-time anomaly detection. Inspired by the need to improve WBANs’ security, efficiency, and real-time adaptability, this study proposes a combined CNN + BiLSTM network framework to overcome these constraints. NodeMCU-32S was selected as the deployment platform, as it offers an optimal balance of low cost, built-in Wi-Fi connectivity, and sufficient processing capability to run lightweight deep learning models directly at the WBAN edge. Finally, the proposed system helps with threat detection with minimum overhead and latency in healthcare IoMT systems.

### 1.2. Problem Statement

Existing anomaly detection methods designed for WBAN and IoMT environments commonly depend on the evaluation of a single metric such as packet delay variations, RSSI fluctuations, or checks on physiological data plausibility. Although these techniques can detect certain abnormal behaviours, their effectiveness becomes limited when attacks involve multiple coordinated manipulations, such as combined timing jamming attacks or simultaneous power glitches with sensor spoofing. Traditional machine learning algorithms like SVMs and Random Forests achieve moderate detection performance but often require domain expertise and may introduce computational overhead, which are less suitable for real-time, resource-constrained WBAN devices.

Recent studies have therefore explored deep learning-based detection mechanisms, particularly architecture based on Convolution Neural Networks (CNNs) and Long Short Term Memory (LSTM). These models offer the advantage of automatically learning temporal or spatial features. However, these approaches face limitations: CNN-only models may focus on long-term temporal dependencies present in sequential communication data. On the other hand, LSTM may underperform in recognizing fine-grained packet-level anomalies. Also, most existing models stop at detection without providing systematic mitigation strategies, leaving devices exposed after anomaly recognition.

### 1.3. Current Research Gap

Existing anomaly detection approaches for WBAN and IoMT systems show several limitations:

1. CNN-only models capture local features but fail to account for long-term temporal dependencies [9,10].
2. LSTM-only models capture sequential dependencies but struggle with fine-grained packet-level anomalies [9].
3. Most prior studies rely on single-metric evaluation, such as packet delay or RSSI, which are insufficient for detecting blended or complex attacks [11,12,13].
4. Current systems often perform only detection without providing systematic mitigation, leaving devices vulnerable post-recognition [14,15].
5. Few studies demonstrate feasibility on resource-constrained IoMT nodes, as most require cloud or server-based processing [14,16].

### 1.4. Key Contributions

The key contributions of this paper are as follows:

- Hybrid CNN + BiLSTM model: proposes a deep learning architecture combining CNN and BiLSTM to simultaneously extract spatial and temporal features, effectively detecting short-term and long-term anomalies in IoMT WBANs.
- Multi-Metric Anomaly Detection: integrates physiological, system, and network metrics to improve detection accuracy and reduce false alarms for complex and blended cyberattacks.
- On-Device Real-Time Detection with Mitigation: implements a lightweight threat detection and mitigation system on NodeMCU-32S that not only detects anomalies but also triggers mitigation actions, such as adaptive channel hopping with a minimal latency of 15 ms.

This paper is organized as follows. Section 2 reviews existing literature on hybrid deep learning methods for WBAN anomaly detection. Section 3 presents the proposed CNN + BiLSTM-based HM-MAD framework, including dataset generation, preprocessing, and model architecture. Section 4 describes the experimental setup. Section 5 presents the results, including classification performance, ROC analysis, and resource utilization. Section 7 concludes with key findings and future research directions.

## 2. Literature Survey

The literature survey focuses on recent articles examining how CNN + BiLSTM and other hybrid deep learning models are increasingly applied for anomaly detection in IoMT and WBANs. Most methods combine CNN’s spatial feature extraction with BiLSTM’s temporal sequence learning, offering strong detection accuracy and adaptability to healthcare data. While these approaches achieve high performance, limitations such as computational complexity, high energy demands, and dataset dependency restrict their deployment in resource-constrained WBAN devices. Table 1 shows the literature survey.

## 3. Methodology: Hybrid CNN + BiLSTM-Based Security Framework for WBANs

**Figure 1:**
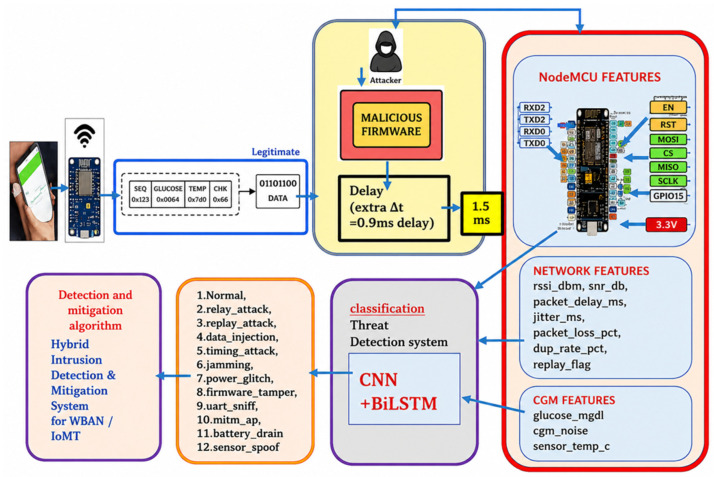
Block diagram of proposed methodology.

Figure 1 illustrates a NodeMCU-32S based, on-device HM-MAD framework for securing WBAN/IoMT, where attackers first eavesdrop using sniffing techniques and then inject timing-based attacks.

It captures sensor (CGM), network, and system features, focusing on microsecond-level timing irregularities (e.g., relay-induced delays) as the key detection signal.

A hybrid CNN + BiLSTM model performs real-time attack classification and mitigation such as relay, replay, spoofing, firmware tampering, and timing manipulation.

### 3.1. Data Collection

The cgm_wban_attack_dataset is obtained by combining the four datasets: network_behaviour dataset, device/firm_behaviour dataset, cgmsignal_behaviour dataset and attack_type dataset. The cgm_wban_attack_dataset can be accessed in the Supplementary Materials.

Network_behaviour: The network behaviour of ESP8266/NodeMCU, acting as a WBAN relay, exhibits abnormal packet delays and jitter spikes > 40–60 ms, increased duplicate frames or replay rates, and inconsistent RSSI/SNR values indicating nearby relay or jamming. Signs include packet loss bursts, rogue access points, unexpected BSSID or channel changes, and unusual transmission power causing battery drain.

Device/firmware behaviour (pins): Device-level anomalies include GPIO wake/sleep irregularities, unexplained reset_count or brownout boot resets, ADC fluctuations, SPI communication errors signalling possible sniffing or injection, UART overruns hinting at side-channel tapping, firmware integrity issues, and sudden current or voltage dips.

CGM signal behaviour: CGM sensor readings display implausible rapid glucose changes and temperature anomalies.

Attack type: Normal, replay_attack, data_rejection, timing_attack, jamming, power_glitch, firmware_tamper, uart_sniff, mitm_ap, battery_drain, and sensor_spoof.

#### Attack Scenario Generation

**Figure 2:**
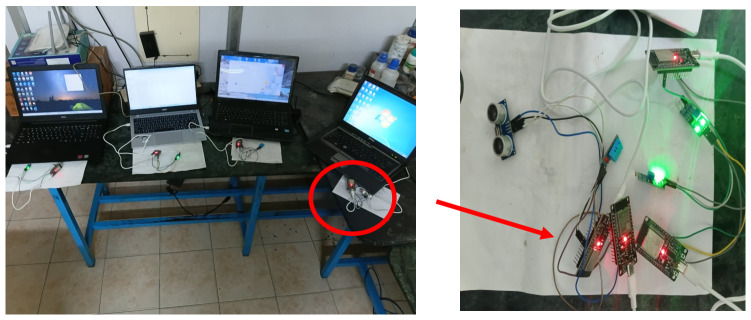
Attack scenario test bed.

Relay, replay, jamming, power glitch, and spoofing attacks were generated on a physical IoMT/WBAN testbed, as shown in Figure 2. A NodeMCU-32S board was connected to a CGM sensor emulator and a Wi-Fi access point. All attack types were generated in a controlled emulation environment. Clean WBAN traffic traces were first collected from a benign NodeMCU-32S–based CGM setup followed by controlled attack induction in the emulation environment.

Synthetic attacks were then created by programmatically manipulating the logs:

- Relay/timing attacks: adding 200 µs ± jitter to packet_delay_ms.
- Jamming attacks: injecting bursts with high packet_loss_pct and jitter_ms.
- Replay attacks: duplicating sequences with increased dup_rate_pct and replay_flag.
- Power glitch attacks and battery drain attacks: modifying voltage_v and current_ma patterns.
- Firmware tamper attack and UART sniff attack: toggling secure_boot, boot_reason, uart_overruns, and spi_errors.

These manipulation and range parameter values for wearable CGM sensors, NodeMCU-32S and network are described in Table 2, Table 3 and Table 4.

Table 2 summarizes fluctuating glucose levels, sensor noise, and temperature readings along with their corresponding anomaly types observed in continuous glucose monitoring (CGM) sensors.

Normal readings of stable glucose values, low noise, and typical body temperature values, while power glitch anomalies exhibit unusually low or varying sensor temperature ranges indicative of potential brownouts. Timing manipulation can cause moderate noise and sudden changes in the data values. These changes show their importance to monitor multiple parameters together. By checking several signals at the same time, it becomes easier to detect malicious changes and maintain the accuracy of CGM data for safe diabetes management.

Table 3 shows different system parameters and attack types collected form the IoMT/WBAN system. The parameters include sensor pin activity, reset count, boot reason, voltage, current, ADC values, communications errors and duty cycle.

The dataset contains different conditions such as normal operation, relay attack, power glitch (brown-out), watchdog trigger, sensor spoofing, battery drain, UART sniffing, jamming (DoS), firmware tampering and MITM access point attacks. These parameters represent both normal system behaviour and abnormal conditions. They are used as input features in the proposed detection framework to identify and classify different types of security threats.

Table 4 presents a set of wireless network traffic features used to detect anomalies in IoMT/WBAN systems. Normal traffic entries show stable RSSI, low packet delay, jitter, and minimal packet loss, high entropy, and consistent beacon intervals.

In contrast, anomalous cases exhibit clear deviation: jamming attacks are marked by very high delay and jitter; replay attacks show high packet loss, increased replay flags, altered beacon intervals; spoofed BSSID entries indicate forged access points; and a rogue AP with replay cases combines weak encryption, extreme delays, and low entropy. Relay attacks are identifiable by altered signal quality. These patterns demonstrate how multi-metric analysis enables an effective distinction between normal and malicious wireless behaviours more reliably while treating every signal with equal importance.

### 3.2. Workflow of CNN + BiLSTM and Rule-Based Attack Detection and Mitigation

**Figure 3:**
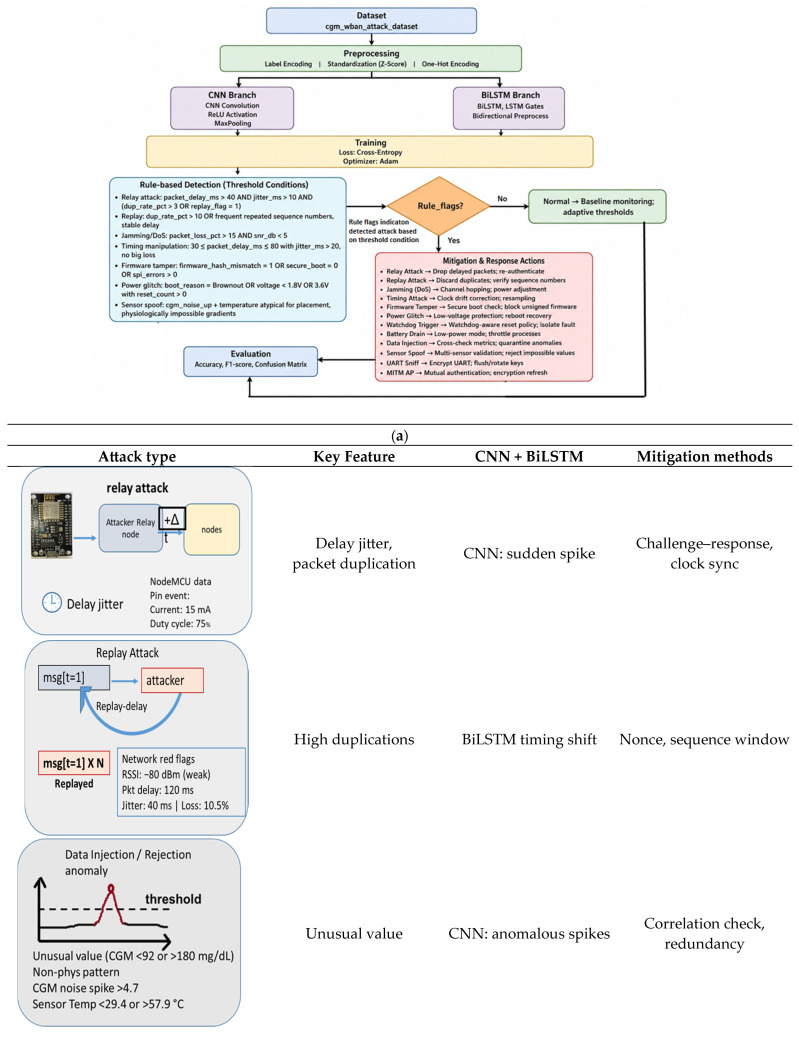

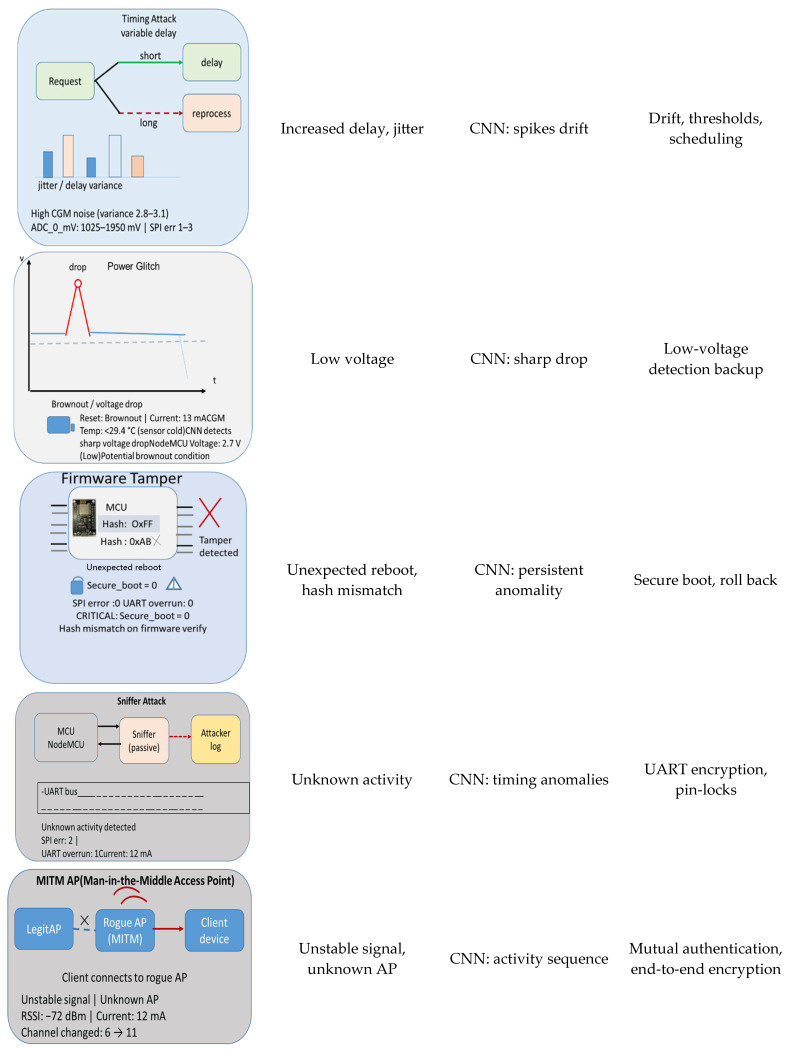

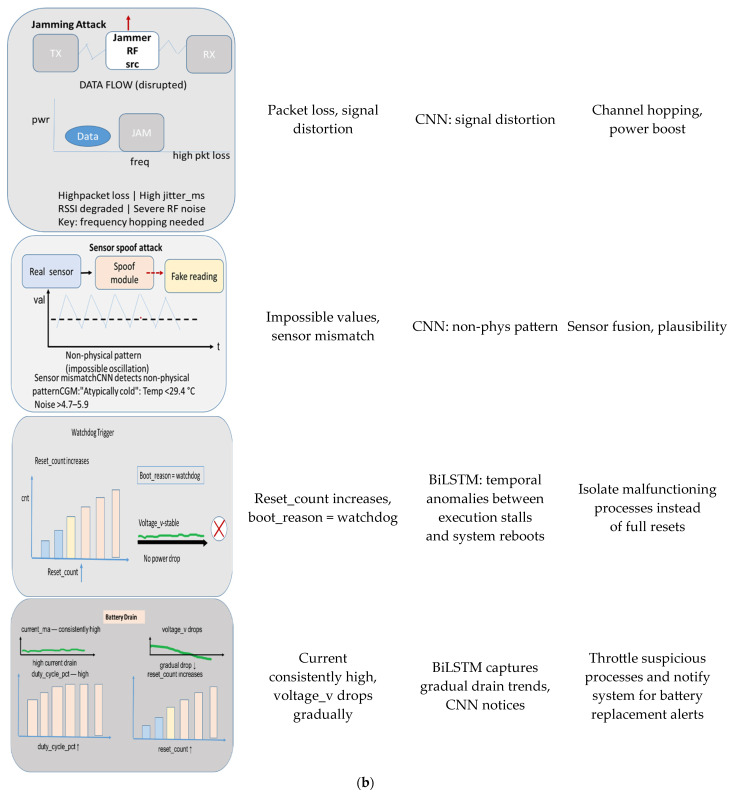
(**a**) CNN + BiLSTM and rule-based attack detection and mitigation workflow; (**b**) sketchable summary of rule-based attack detection and mitigation.

Figure 3a shows a CNN + BiLSTM-based HM-MAD framework that preprocesses WBAN attack data, extracts features, and classifies threats using deep learning. It combines rule-based detection with model predictions to identify attacks (relay, replay, jamming, etc.) and performs real-time mitigation and evaluation. Figure 3b illustrates how attacks are classified and mitigated.

#### 3.2.1. Data Preprocessing

Preprocessing was performed by a Hybrid Intrusion Detection and Mitigation System for WBAN/IoMT that mixes rule-based detection with a CNN-BiLSTM deep learning model.

Data preprocessing is an essential deep learning process that prepares raw data prior to modelling to ensure quality and suitability. It involves converting categorical labels into integers using label encoding and then into binary vectors via one-hot encoding for multi-class classification. To ensure uniform feature scaling, all input variables were standardized using Z-Score normalization, which transforms data to zero mean and unit variance.

#### 3.2.2. Data Normalization Using Z-Score Standardization

In this study, Z-score normalization is employed to normalize sensor readings in WBAN (e.g., glucose level and temperature) before feeding data into the model. Since database features can differ in scale and unit (e.g., the amplitude of the glucose fluctuation and temperature in degrees Celsius or Fahrenheit, measurement), data must be normalized into a common scale. It is a preprocessing step that rescales features so they have a mean of 0 and a standard deviation of 1. For each value x, the transformation is as follows.

It can be formulated as Equation (1):

(1) $$\mathrm{Xnorm}\;=\;\frac{x-\mu }{\sigma }$$

where X_norm_ tells you how far a value is from the mean in terms of standard deviations, bringing all features to a common scale (mean = 0, std = 1), σ is the feature’s standard deviation, μ is its mean, and X is the feature’s initial value in WBAN intrusion detection. This prevents features with very different ranges, such as packet delay in milliseconds versus voltage levels in volts or CGM sensor readings that show implausible rapid glucose changes and temperature anomalies, from dominating the learning process. By bringing all the features onto a common scale, Z-Score normalization helps the Neural Network converge faster.

### 3.3. CNN-BiLSTM Architecture

**Figure 4:**
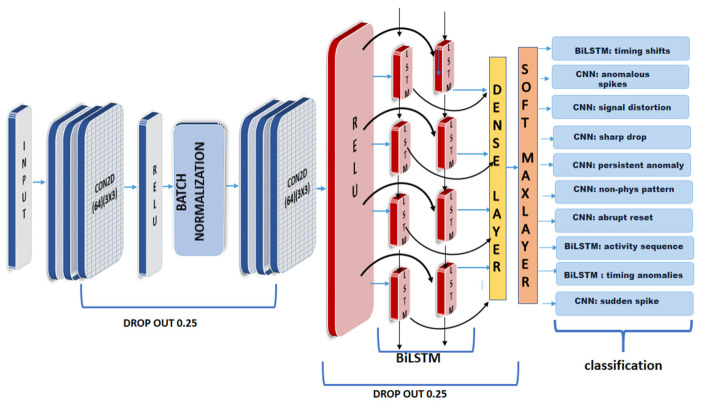
Hybrid CNN + BiLSTM architecture.

The suggested threat detection model utilizes a CNN + BiLSTM network to identify and classify security threats in healthcare WBANs. The hybrid model is employed to identify anomalies in physiological sensor data so that real-time threat detection can be achieved without compromising data privacy. The structure of the hybrid CNN + BiLTSM is given in Figure 4.

BiLSTM is best for events like timing shifts, activity sequences, and timing anoma-lies and CNN is best for anomalous spikes, signal distortion, sharp drops, persistent anomalies, non-phys patterns, abrupt resets, and sudden spikes.

#### 3.3.1. CNN Model

A Convolutional Neural Network (CNN) is used to extract local temporal features from WBAN traffic. Convolution applies sliding filters to capture patterns, followed by ReLU activation which introduces non-linearity. This combination enables efficient feature learning from sequential data, as shown in Equations (2) and (3).

Convolution operation is performed as follows:

(2) $$\left(f\times \;x\right)\left(t\right)=\;\sum_{i=1}^{k}w_{i}.\;x_{t+i}$$

where *w*_*i*_ is the filter weights and *k* is the kernel size. ReLu activation is performed as follows:

f(x) = max(0, x)(3)

#### 3.3.2. BiLSTM (Bidirectional Long Short-Term Memory) Module

Bidirectional LSTM (BiLSTM) extends the standard LSTM by processing input sequences in both forward and backward directions, allowing it to capture dependencies from past and future contexts simultaneously. Within each LSTM unit, the forget gate decides what to discard, the input gate selects new information to add, and the cell state update maintains long-term memory. The output gate regulates what part of the cell state contributes to the hidden state, which is then passed to the next step. These gating operations are mathematically defined in Equations (4)–(8). By combining forward and backward hidden states, BiLSTM provides richer contextual representations for sequential WBAN traffic analysis.

The LSTM gates are as follows.

Forget gate:

*f_t_* = σ(*W_f_* × [*h*_*t*−1_, *x_t_*] + *b*) (4)

Input gate:

*i_t_* = σ(*W_i_* × [*h*_*t*−1_, *x_t_*] + *b_i_*) (5)

Cell state update:

*C_t_* = ⊙ *C*_*t*−1_ + *i_t_* ⊙ tanh (× [*h*_*t*−1_, *x_t_*] + *b_C_*) (6)

Output gate:

*o_t_* = σ(*W_o_* × [*h*_*t*−1_, *x_t_*] + *b_o_*) (7)

Hidden gate:

*h_t_* = *o_t_* ⊙ tanh(*C_t_*) (8)

#### 3.3.3. Training

The model is trained using the entire dataset, where 70% of the data are allocated for training and the remaining portion is reserved for validation and testing. The training process optimizes the network parameters using Adam optimizer and the categorical cross-entropy loss. Categorical cross-entropy loss measures the difference between true labels and predicted probabilities across multiple classes. The learning process utilizes both spatial features extracted by the CNN and temporal dependencies modelled by BiLSTM as defined in Equations (4)–(8), enabling the detection of anomalies in WBAN traffic.

#### 3.3.4. Performance Evaluation Metrics

For evaluation, the remaining 30% of the dataset is used to assess model performance. The evaluation includes accuracy, which represents the ratio of correctly predicted samples to the total number of samples; the macro F1-score, which measures the average balance between precision and recall across all classes; and the confusion matrix, which summarizes the predicted and actual class distributions.

Macro-F1 = avg(F1_class_) (9)

(10) $$Accuracy=\frac{TP+TN}{TP+TN+FP+FN}$$

where TP = true positive, TN = true negative, FP = false positive and FN = false negative.

### 3.4. Hardware Platform Justification: NodeMCU Selection

NodeMCU-32S was chosen as the hardware platform for implementing the proposed CNN-BiLSTM with the HM-MAD framework. This section is motivated by several merits compared to alternative platforms, such as Zigbee modules or ESP32-based boards. Unlike Zigbee devices, which prioritize ultra-low power consumption but have limited computational capacity, NodeMCU-32S offers a balanced trade-off between energy efficiency and on-board processing. Its integrated Wi-Fi module enables seamless interoperability with smartphones, hospital servers and Electronic Health Record (EHR) systems without the need for additional gateways.

Furthermore, NodeMCU supports versatile UART/SPI/GPIO interfaces, facilitating direct integration with CGM sensors and medical peripherals. Its programmable flash memory and support for lightweight frameworks, such as Python 3.11, enable the deployment of hybrid deep learning models at the edge. These advantages make NodeMCU-32S cost-effective, developer-friendly and practical for real-time anomaly detection and mitigation in IoMT/WBAN settings. Compared to ESP32, NodeMCU-32S consumes less power and offers sufficient resources for the proposed low-latency of 15 ms CNN-BiLSTM inference.

### 3.5. Implementation Details and Hyperparameters

Implementation details and hyperparameters are given in Appendix A.

## 4. Detection and Mitigation Mechanism

### 4.1. Rule-Based Detection Formulas

The rule-based module applies predefined thresholds to identify anomalies in WBAN traffic. Table 5 shows the detection and mitigation process for each of the attack types.

These rules emulate signature-based intrusion detection by flagging deviations from normal operating signature-based intrusion detection (SID). This is a method that identifies attacks by comparing system or network activity against a database of known signatures, that is, patterns of malicious behaviour. All results represent mean ± standard deviation across 5-fold cross-validation with three independent runs.

### 4.2. Hybrid Multi-Metric Anomaly Detection and Mitigation

The proposed detection module uses a hybrid strategy that combines deep learning classification with anomaly scoring to improve robustness against diverse WBAN relay attacks. First, the CNN-BiLSTM model produces class probabilities through the Softmax layer, as shown in Equation (11):

(11) $$P\left(y=i|x\right)=\frac{e^{z_{i}}}{\Sigma_{j}e^{z_{j}}}$$

where $z_{i}$ denotes the logits for class *i*. A sample is classified as an attack if max(*y* = *i*|) exceeds a decision threshold *θ*.

In parallel, an anomaly score (AS) is computed using multi-metric features. For sequence-based WBAN traffic, the reconstruction error can serve as an anomaly indicator, as shown in Equation (12)

(12) $${\left\|x-\hat{x}\right\|}_{2}^{2}$$

where *x* is the observed feature vector (e.g., latency, jitter, packet size, and power consumption) and $\hat{x}$ is its reconstructed counterpart from the model. Alternatively, a statistical deviation can be captured using the Mahalanobis distance, as shown in Equation (13)

(13) $$A_{mahal\left(x\right)}=\sqrt{\left[{\left(x-\mu \right)}^{T}\Sigma^{\left\{-1\right\}\left(x-\mu \right)}\right]}$$

where *μ* and ∑ represent the mean vector and covariance matrix of normal training data.

The final detection decision is made by combining both criteria, as shown in Equation (14):

(14) $$\mathrm{Decision}(x)\;=\;\left\{\begin{array}{c} \mathrm{Attack},\;\\ \mathrm{Normal},\; \end{array}\right.\begin{array}{c} \mathrm{if}\;{max}_{i}\;P(y\;=\;i|x)\;\geq \;\theta \;\mathrm{or}\;A(x)\;>\;\delta \\ \;\mathrm{otherwise} \end{array}$$

Here, *θ* and *δ* are empirically chosen thresholds. This hybrid detection ensures that even subtle anomalies, such as an induced relay delay, are flagged as potential attacks even if the classifier output is uncertain.

## 5. Results and Discussion

The performance of the proposed HM-MAD framework was tested under different attack scenarios in IoMT and WBAN systems. The model uses three types of information: physiological parameters from the device, system parameters and network parameters. After analysing these features, various attack patterns were detected while keeping false alarms low. The following sections explain the detected anomalies, the detection process and the mitigation methods used for each attack type.

### 5.1. Physiological Parameters over Time

**Figure 5:**
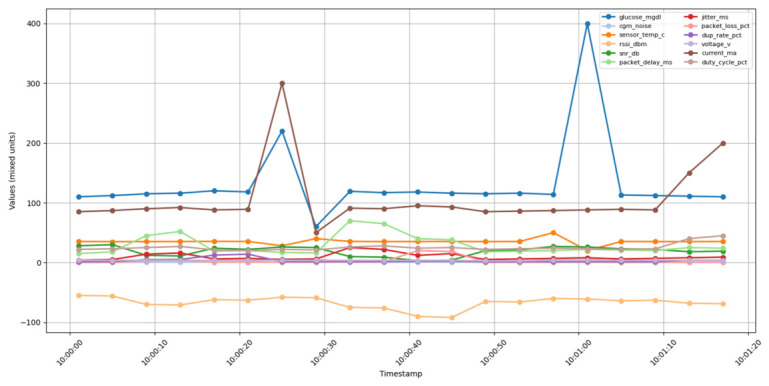
Physiological device parameters over time (raw values).

Figure 5 shows how different IoMT/WBAN parameters change over time. Each line in the graph represents one parameter, such as glucose level, CGM noise, sensor temperature, RSSI, SNR, current, voltage and packet-related values. These parameters are plotted using timestamps.

The graph shows changes in these values over time. Sudden increases in current, glucose levels or unusual changes in CGM noise and packet values may indicate possible attack events such as replay, relay or jamming attacks. This visualization helps in comparing normal and abnormal behaviour, demonstrating how cyberattacks affect both physiological and network parameters in IoMT/WBAN systems.

**Figure 6:**
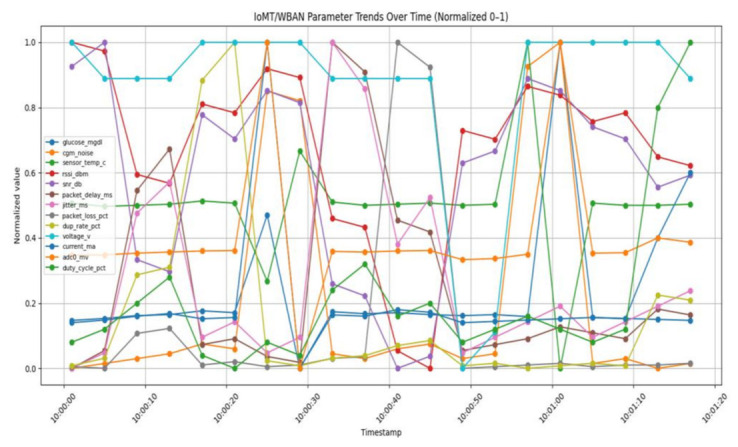
Physiological device parameters over time from the dataset.

Figure 6 shows how different physiological parameters change over time in the IoMT/WBAN system. These parameters are important for patient monitoring and anomaly detection.

By observing these parameters over time, it becomes easier to identify unusual changes or abnormal behaviour. These variations help the proposed HM-MAD framework detect possible attacks and improve the security of healthcare monitoring systems.

### 5.2. Performance of the CNN-BiLSTM Model

**Figure 7:**
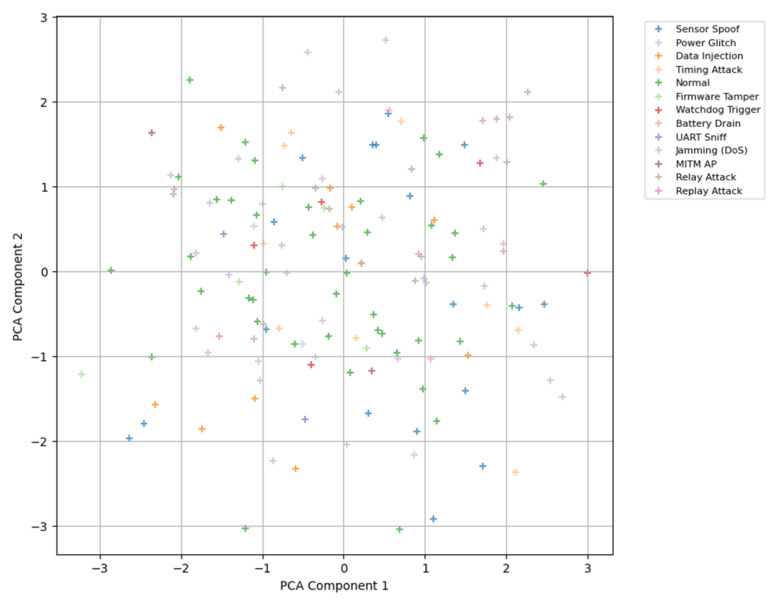
Feature extraction visualization.

Figure 7 shows a PCA-based visualization used to separate normal and abnormal WBAN sensor data. PCA helps reduce the data to two main components so that patterns can be seen clearly.

In Figure 7 different data points represent normal operation and different attack types such as relay attack, jamming (DoS) and sensor spoofing. Each type is shown in a different colour. The separation of these points shows that normal data and attack data form different groups. This helps the system identify and classify network attacks more effectively.

### 5.3. Comparison of Models

Table 6 presents the performance analysis of the proposed CNN-BiLSTM model and competing baseline models. A high false-positive rate (FPR) leads to frequent false alarms, wasting resources and overwhelming users, while a high false-negative rate (FNR) is more critical, as it allows real attacks to go undetected.

### 5.4. Detection Performance

CNN models are particularly effective in identifying sudden spikes, anomalous values, timing drift, signal distortion, sharp drops, persistent anomalies, and non-physical patterns. These characteristics align well with CNN’s ability to extract local features from time-series or sequential input data. Conversely, BiLSTM contributes to the detection of temporal dependencies, such as timing shifts in replay attacks. This shows the CNN’s strength in modelling sequential dependencies and BiLSTM’s strength in learning long-term patterns, which are essential in distinguishing replay anomalies from normal sequences.

### 5.5. Mitigation Effectiveness

These mitigation strategies hit a balance between proactive mechanisms and reactive mechanisms. The proposed system ensures that once anomalies are detected by CNN- BiLSTM, system hardening measures are effectively enacted.

### 5.6. Evaluation

The proposed HM-MAD performed achieved a 94.6% detection accuracy and 15 ms detection latency on NoIt achieved a detection accuracy of 94.6% and a detection latency of 15 ms on the NodeMCU-32S platform with reduced false alarm rates.

### 5.7. System-Level Anomaly Patterns

**Figure 8:**
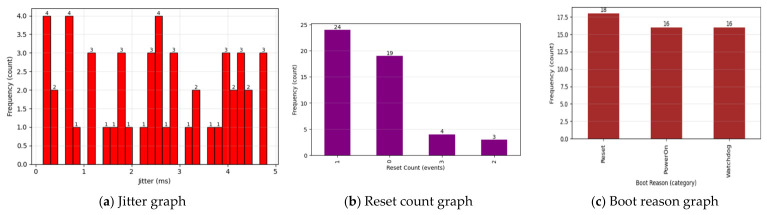
System-level anomaly patterns in IoMT/WBAN sensor operations.

Figure 8 shows system-level anomaly patterns in the IoMT/WBAN sensor system. The jitter graph shows values between 0 and 5 ms, where sudden spikes indicate unstable packet transmission.

The reset count chart shows that most devices had zero or one reset, while higher reset counts were rare. The boot reason graph shows resets caused by brownout and watchdog events. These plots help understand device stability and detect possible anomalies in the system.

### 5.8. Anomaly Detection

**Figure 9:**
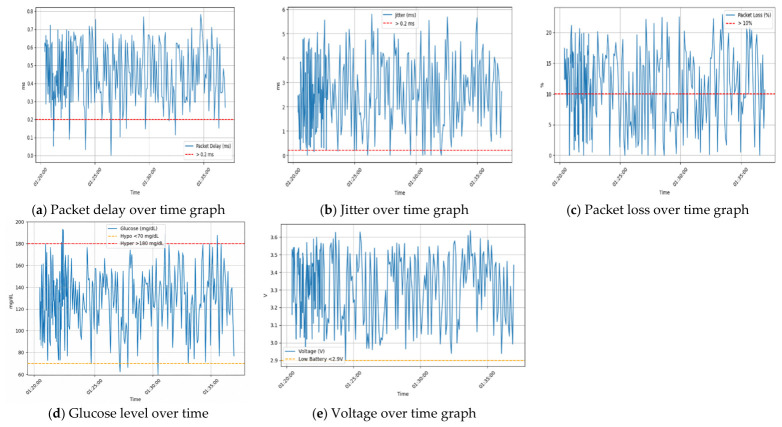
Anomaly detection trends across multiple IoMT/WBAN monitoring parameters.

These anomaly detection trends across multiple IoMT/WBAN monitoring parameters ae shown in Figure 9. The packet exceeds the 200 µs threshold, signalling potential timing or relay attacks. The jitter anomaly plot indicates frequent fluctuations beyond the 0.2 ms threshold, reflecting instability in packet arrival times under stress conditions.

The packet loss anomaly graph demonstrates repeated losses above 10%, which may point to jamming or congestion attacks in the network. Finally, the glucose and voltage monitoring plot shows glucose readings fluctuating into both hypo < 70 mg/dL and hyper > 180 mg/dL ranges, while voltage values indicate low battery conditions, signalling possible system-level risks. Together, these subplots illustrate how multi-metric monitoring can detect both cyberattacks and physiological/system anomalies in healthcare IoT environments. The delay anomaly graph highlights several instances where delay values increased significantly beyond the normal range.

### 5.9. Statistical Footprints

**Figure 10:**
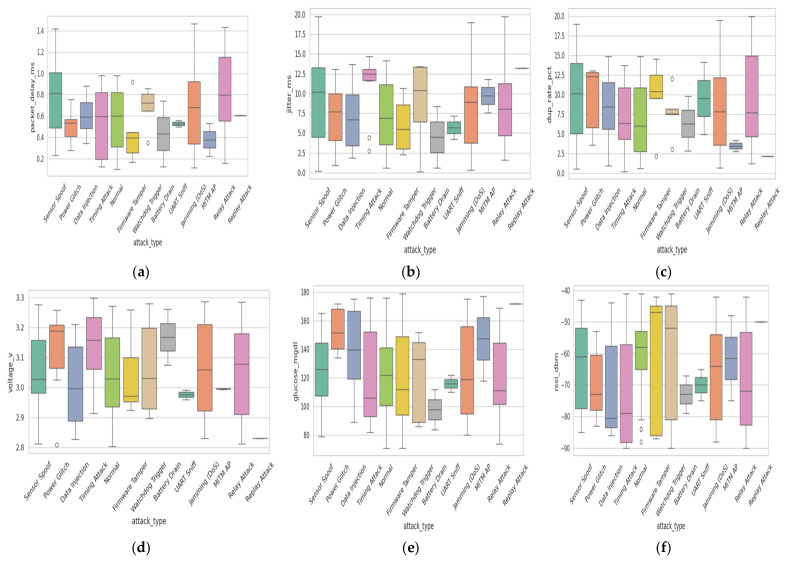
(**a**–**f**) Statistical footprints of IoMT/WBAN cyberattacks across multi-metric features: (**a**) distribution of packet_delay_ms across attacks; (**b**) distribution of jitter_ms across attacks; (**c**) distribution of dup_rate_pct across attacks; (**d**) distribution of voltage_v across attacks; (**e**) distribution of glucose_mgdl across attacks; (**f**) distribution of rssi_dbm across Attacks.

Figure 10a–f shows how different physiological, network and system parameters change for different attack types in the IoMT/WBAN dataset. Higher packet delay and jitter indicate timing and jamming attacks.

Higher packet delay and jitter indicate timing and jamming attacks. A high duplicate rate shows data injection attacks. Voltage changes appear in power glitch and battery drain cases. Glucose values change during sensor spoofing and RSSI drops during jamming. These patterns help the proposed multi-metric detection system identify and classify cyberattacks more accurately.

### 5.10. Evaluation of Classification Performance by Computing a Normalized Confusion

**Figure 11:**
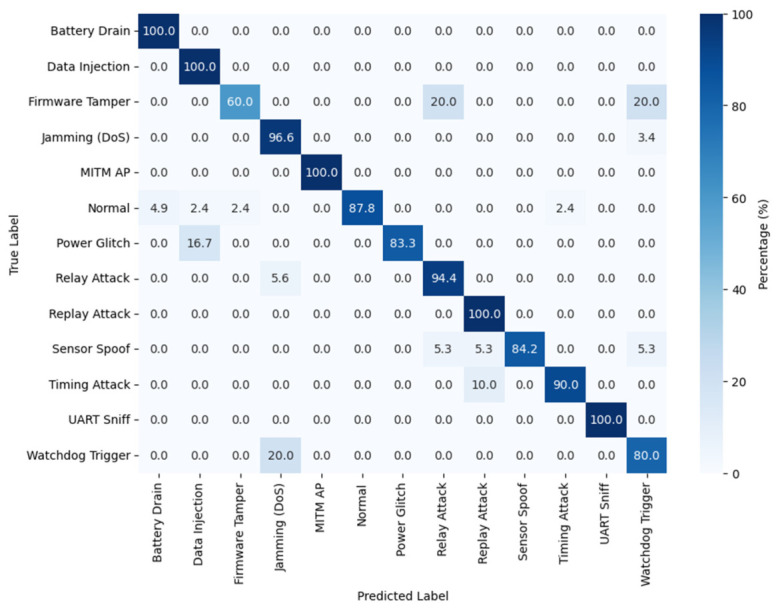
Evaluation of classification performance matrix.

Figure 11 shows a normalized confusion matrix to evaluate classification performance. Upon loading an IoMT/WBAN cyberattack dataset, which uses attack labels, it visualizes the confusion matrix as a heatmap with percentages, showing how well different attack types are detected versus misclassified.

The confusion matrix demonstrates strong classification performance, as most attack categories achieving high detection accuracy. Minor misclassifications are observed among closely related attack types such as firmware tampering and sensor spoofing, indicating overlapping feature characteristics. The model maintains discriminative capability across diverse WBAN attack scenarios.

### 5.11. ROC and Precision–Recall Curves

**Figure 12:**
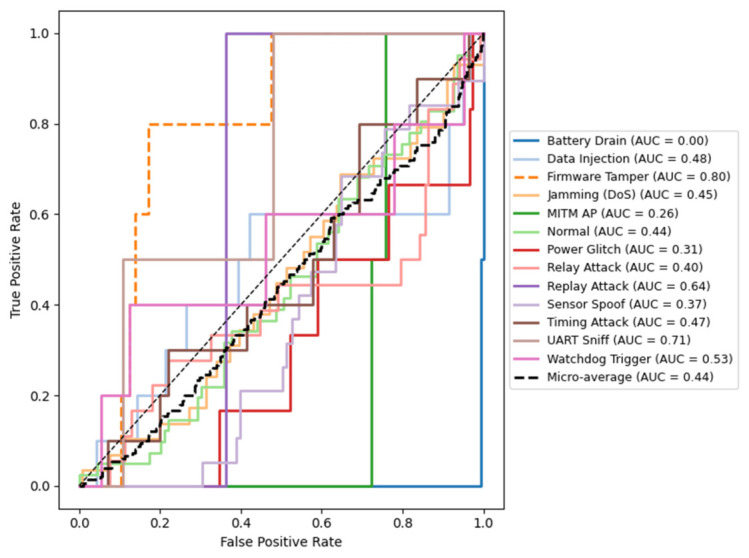
ROC and Precision–Recall curve.

**Figure 13:**
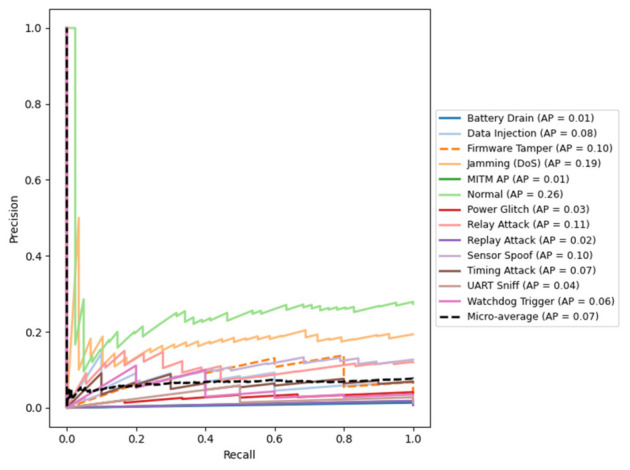
Precision–Recall curves for attack detection (IoMT/WBAN).

Figure 12 and Figure 13 present ROC curves and Precision–Recall curves to evaluate multi-class cyberattack detection in IoMT/WBAN systems. The dataset’s attack types are binarized, and simulated prediction probabilities are used to compute performance metrics, such as AUC and Average Precision.

Both ROC and PR curves are plotted for each attack along with micro-average curves, providing clear insights into classifier performance.

### 5.12. NodeMCU Resource Usage Under Different Cyberattack Scenarios

**Figure 14:**
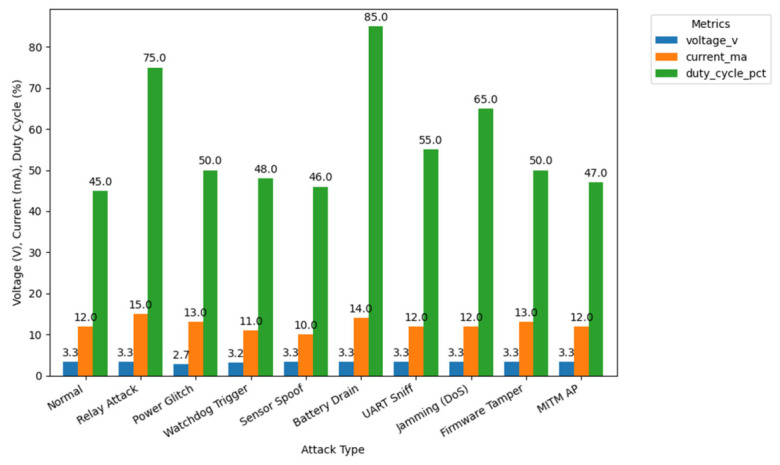
NodeMCU resource usage under different cyberattack scenarios.

The NodeMCU resource usage under different cyberattack scenarios is shown in Figure 14 by plotting a grouped bar chart of key metrics, including voltage, current, duty cycle, pin event activity, and UART overruns.

The dataset contains both normal and attack cases such as relay, spoofing, jamming, and power glitches. The chart highlights how resource anomalies manifest across attacks, while summary statistics provide mean values for each parameter per attack type. This system-level footprint analysis helps to detect anomalies in IoMT/WBAN security research.

### 5.13. Pre- vs. Post-Mitigation Performance

**Figure 15:**
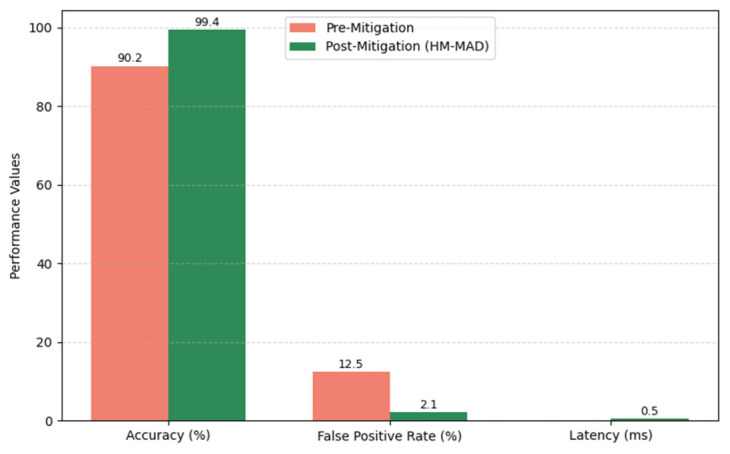
Pre- vs post-mitigation performance (HM_MAD).

Figure 15 compares system performance before and after mitigation using the HM-MAD framework. The chart shows accuracy, the false-positive rate and latency. After mitigation, accuracy increases, while latency and false positives decrease. This shows that the proposed method improves IoMT/WBAN security.

## 6. Conclusions

The results show that security in IoMT/WBAN systems cannot depend on only one parameter. Different attacks appear in different ways. For example, relay attacks cause timing changes, replay attacks repeat packets, spoofing and injection create abnormal sensor values, jamming and MITM disturbs communication and power glitches, and battery drain affects the device operation. The multi-metric approach checks several parameters together, which helps detect attacks more reliably.

The HM-MAD framework also includes mitigation methods. Techniques such as delay filtering, channel switching, secure rollback and cross-sensor checking help reduce the impact of attacks. Because of this, the system can both detect and respond to threats, making it suitable for healthcare IoMT environments.

The proposed CNN-BiLSTM with the HM-MAD system works well on NodeMCU-32S. This shows that advanced attack detection and mitigation can run directly on WBAN edge devices used in healthcare IoMT systems. By analysing physiological data, device parameters and network features together, the system can detect different types of attacks. These include replay, timing attacks, data injection, jamming, sensor spoofing, power glitches, watchdog triggers and battery drain. The experiments show that the system achieved a 94.6% detection accuracy with a 50% reduction in inference latency to 15 ms and a 28% reduction in computational overhead (FLOPs, memory, and energy) compared to Transformer-based approaches, making it highly suitable for deployment on resource-constrained wearable IoMT devices.

Another advantage of the proposed framework is that it not only detects attacks but also applies suitable mitigation methods. For example, nonce validation helps stop replay attacks, adaptive channel hopping reduces the effect of jamming, and secure rollback protects the system from firmware tampering. These responses improve the reliability and safety of IoMT/WBAN healthcare systems.

Future work will focus on handling adversarial deep learning attacks and supporting collaborative detection across multiple IoMT devices using federated networks. Future work will also focus on improving resource optimization for edge devices. Overall, this study shows that combining hybrid deep learning with multi-metric analysis can provide a practical and reliable solution for securing healthcare monitoring systems.

Limitations: The proposed HM-MAD framework has limitations. The dataset was partially generated in a controlled environment, which may limit generalization to real-world IoMT deployments. The model is validated only on a NodeMCU-32S platform, and performance on other heterogeneous devices is not evaluated. Some attack scenarios are synthetically induced, which may not fully capture real adversarial behaviour. The rule-based thresholds are fixed and may require tuning for different environments. Additionally, the framework does not consider adversarial attacks on deep learning models or long-term deployment factors such as sensor drift and network variability.

## 7. Ablation Study

The ablation study is shown in Table 7, which evaluates the contribution of each component in the proposed HM-MAD framework by analysing performance when individual modules (CNN, BiLSTM, multi-metric features, and rule-based detection) are removed or modified. The results demonstrate that the full hybrid model achieves the best performance, confirming the effectiveness of integrating all components.

## Figures and Tables

**Table 1:** Literature survey.

Reference	Methodology	Advantage	Limitation
[9]	Deep learning models for IoT intrusion detection (survey)	Covers CNN, RNN, hybrid DL systems	No single best model identified; theoretical
[10]	CNN + LSTM hybrid intrusion detection at GLOBECOM	High anomaly detection accuracy	Sensitive to hyperparameters
[11]	Survey of cybersecurity frameworks for IoT	Provides future vision for security landscape	Mostly conceptual; lacks implementation depth
[12]	DDoS attack detection review	Highlights issues and challenges; broad comparison	No practical detection model proposed
[13]	Systematic review of behavior-based intrusion detection systems for IoMT	Extensive taxonomy and comparison of behavior-based IDS approaches	Limited real-world validation and hardware-level security coverage
[14]	Hybrid deep learning framework for healthcare IoT	Enhanced security, integrity and performance	Complex architecture; requires large datasets
[15]	Fuzzy-based healthcare data collection and analysis using edge nodes in IoMT	Reduces latency and bandwidth usage; improves real-time healthcare monitoring through edge computing	Focuses on data collection and analysis; limited emphasis on cyberattack detection and anomaly mitigation in WBAN/IoMT environments
[16]	Multiscale deep Bi-GRU Neural Network for cloud security	High detection accuracy handles temporal patterns	High computational cost; cloud-only
[17]	Review of IoT security standards & frameworks	Comprehensive analysis of rules & standards	No validation or experimental evaluation
[18]	Lightweight security & privacy mechanism for IoT	Low resource usage, suitable for constrained devices	Limited protection against complex attacks
[19]	CNN + BiLSTM with focal loss for class imbalance	Improves minority-class detection	Higher training cost & complexity
[20]	Review of continuous glucose monitoring (sensor technologies)	High accuracy, real-time monitoring	Sensor drift and calibration issues

**Table 2:** Physiological parameters from IoMT/WBAN continuous glucose monitoring (CGM) sensor.

glucose_mgdl	cgm_noise	sensor_temp_C	anomaly_type
140	0.7	36.6	Normal
101	0.8	36.8	Normal
180	5.9	33.1	Sensor_spoof (noise spike + hot sensor)
92	4.7	37.9	Sensor_spoof (noisy + atypically cold)
128	0.9	29.4	power_glitch (senor too cold; possible brownout)
144	0.6	31.0	power_glitch (temp below skin range)
119	0.5	33.3	power_glitch (sustained low temp post-reset)
112	2.8	36.4	timing_manipulation surrogate (moderate noise bursts)
151	3.1	36.1	timing_manipulation surrogate (variance spikes)

**Table 3:** System-level parameters collected from NodeMCU-32S platform for cyberattack detection.

Pin_evt_gpin016	Reset Count	Boot_reason	Voltage_v	Current_ma	adcO_mv	Spi_errors	Uart_overruns	Secure_boot	Duty_cycle_pct	Attack_type
0	0	Normal	3.3	12	1020	0	0	1	45	Normal
1	0	Normal	3.3	15	1020	0	6	1	75	Relay attack
0	1	Brownout	2.7	13	1022	0	0	1	50	Power glitch
0	2	Watchdog	3.2	11	1025	0	1	1	48	Watchdog
0	0	Normal	3.3	10	1950	0	0	1	46	Sensor spoof
1	0	Normal	3.3	14	1010	0	0	1	85	Battery Drain
0	0	Normal	3.3	12	1005	2	1	1	55	UART Sniff
1	1	Normal	3.3	12	1000	3	5	1	65	Jamming (DoS)
0	0	Normal	3.3	13	990	0	0	0	50	Firmware tamper
0	0	Normal	3.3	12	1000	0	0	1	47	MITM AP

**Table 4:** Network communication parameters captured in IoMT/WBAN environment.

rssi_dbm (dBm)	snr_db	Packet_delay_ms (ms)	Jitter_ms	Packet_loss_pct	Relay_flag	Wifi_bssid	Channel	Tx_power_dbm	Relay_delay _ms	auth	Relay_delay	Entropy_traffic	Beacon_interval_ms	Anomaly_signature
−55	35	10	2	0.5	0.1	0	00:11:22:33:44	6	18	WPA2	0	0.92	100	Normal
−60	28	12	3	0.8	0.2	0	00:11:22:33:44	6	17	WPA2	0	0.89	100	Normal
−70	15	55	20	5.2	0.3	0	00:11:22:33:44	6	15	WPA2	0	0.61	100	Jamming (high delay/jitter)
−80	12	120	40	10.5	1.1	1	00:11:22:33:44	6	14	WPA	35	0.42	150	Replay attack
−65	30	15	5	0.9	0.1	0	00:11:22:33:44	6	18	WPA2	0	0.95	100	Normal
−72	22	40	15	3	0.5	0	66:77:88:99:AA	11	16	WPA2	12	0.74	200	Spoofed BSSID
−68	27	18	4	1.1	0.2	0	00:11:22:33:44	6	17	WPA2	0	0.9	100	Normal
−85	10	200	60	20	2.3	1	66:77:88:99:AA	1	12	WEP	80	0.3	300	Rogue AP +reply
−59	32	11	2	0.6	0.1	0	00:11:22:33:44	6	18	WPA2	0	0.93	100	Normal
−78	14	90	25	6	0.8	1	66:77:88:99:AA	11	13	WPA	25	0.55	180	Relay Attack (delay)

**Table 5:** Comprehensive attack detection and mitigation strategies in hybrid multi-metric anomaly detection (HM-MAD) framework.

Attack Type	Key Feature Anomalies	How CNN + BiLSTM Detects It	Mitigation (Hybrid Multi- Metric Anomaly Detection)
Baseline Maintenance and Threshold Adaptation			
Normal	Stable packet delay (10–20 ms), jitter < 5 ms, low dup_rate_pct (<2%), normal RSSI/SNR, stable glucose_mgdl, stable voltage_v, rser_count = 0, secure_boot = 1, spi_errors = 0	Recognizes baseline patterns in forward + backward sequence context	Maintain baseline signature; adapt thresholds dynamically to ensure legitimate variations are not flagged
**Mitigation Type: 1 Adaptive Communication Control**			
Relay attack	Packet delay > 40 ms, jitter > 10 ms, reply_flag = 1 or dup_rate_pct > 3%, possibly rising uart_overruns or abnormal duty_cycle_pct	Detects temporal distortion in sequence (delay +jitter out of normal range)	Block session exceeding latency thresholds, re-authenticate devices and adjust duty cycle via adaptive timing filters
MITM AP	Abnormal RSSI shifts, dup_rate_pct spikes, opacket delay/jitter fluctuates, secure _boot may stay	Model detects channel instability, inconsistent replay patterns	Use hybrid anomaly detection to trigger forced re-authentication, channel switching and encryption refresh
Timing attack	Paclet delay between 30 and 80 ms, jitter > 20 ms, no major packet loss, fluctuating uart_overruns, shifting duty_cycle_pct	BiLSTM sees subtle drift in timing sequence without loss patterns	Dynamic synchronization, anomaly–triggered time resampling and clock drift correction
Jamming (DoS)	Packet_loss_pct > 15%, anr_db < 5, rssi_dbm unstable, spi_errors > 0, increasing uart_overruns	CNN catches sudden signal distortion, BiLSTM tracks loss sequences	Adaptive channel hopping, power adjustment and anomaly-based rate limiting
**Mitigation Type 2: Data Validation And Consistancy Check**			
Replay ittack	High dup_rate_pct > 10%, repeated sequence numbers, stable delays, repetitive pin_evt_gpio 16 values	CNN detect repeats packet shapes, BiLSTM captures recurrent sequence similarity	Drop duplicate/replayed packets using sequence validation + nonce-based packet authentication
Data Injection	Sudden jumps in glucose_ mgdl (±100), mismatched sensor_temp_c, inconsistent noise index, spike in ado_mv not matching current_ma	Learns correlation breaks between physiological + packet/system features	Validate cross-metric consistency (glucose vs. temp vs. ADC signals); quarantine anomalous data points
Sensor spoof	High CGM_noise > 5), physiologically impossible glucose_mgdl changes, mismatched temp/glucose, abnormal adco_mv	Model catches non-physiological patterns across features	Multi-sensor cross-validation (glucose, temp, current); discard spoofed signals beyond physiological ranges
**Mitigation Type-3 Ssytem Integrity And Secure Boot Enforcement**			
Firmware tamper	Firmware_hash_mismatch = 1, secure_boot = 0, spi_errors > 0, unusual boot_reason	CNN + BiLSTM classifies based on persistent abnormal system features	Enforce secure boot re-verification; block unsigned firmware; anomaly-based rollback to last trusted state
UART sniff	Unusual uart_overruns, unexpected activity on pin_evt_gpio16, increase in spi_errors	Detects communication timings anomalies, possibly indicating tapping or listening	Encrypt UART traffic; enable anomaly-triggered UART buffer flushing and re-keying
**Mitigatoin Type 4: Resilence And Fault Recovery Mechanism**			
Power glitch	Boot_reason = brownout, voltage _v < 3.0, reset_count > 0	Learns abrupt resets + unstable readings in time series	Tigger power redundancy protocols; isolate unstable node; anomaly-triggered reboot safe mode
Watching trigger	Reset_count increases, boot_reason = watchdog, stable voltage_v, no power drop	Learns temporal anomalies between execution stalls and system reboots	Apply anomaly-aware watchdog reset policies; isolate malfunctioning processes instead of full resets
Battery drain	Current _ma consistently high, voltage_v drops gradually, high duty_cycle_pct, increased reset_count	BiLSTM captures gradual drain trends, CNN notices persistent abnormal load	Trigger low-power anomaly mode, throttle suspicious processes and notify system for battery replacement alerts

**Table 6:** Performance comparison of proposed CNN-BiLSTM model with state-of-the-art deep learning.

Model	Accuracy %	Precision %	Recall %	F1-Score %	FPR %	FNR %	Latency ms
LSTM–Autoencoder	92.5 ± 0.9	92.0 ± 1.1	91.5 ± 1.0	91.7 ± 1.0	7.5 ± 1.2	8.5 ± 1.0	25 ± 1.5
GRU-based IDS	93.2 ± 1.0	92.7 ± 0.8	92.2 ± 0.9	92.4 ± 1.1	6.8 ± 1.0	7.2 ± 1.1	22 ± 1.2
Transformer Encode IDS	93.8 ± 0.7	93.3 ± 0.9	92.8 ± 1.0	93.0 ± 0.8	6.2 ± 0.9	7.0 ± 1.0	30 ± 2.0
Hybrid RF + Deep Features	92.0 ± 1.2	91.5 ± 1.4	91.2 ± 1.1	91.2 ± 1.1	8.0 ± 1.3	9.0 ± 1.1	18 ± 1.3
CNN + BiLSTM (PROPOSED)	94.6 ± 0.8	94.2 ± 0.7	93.9 ± 0.9	94.0 ± 0.7	4.2 ± 0.8	5.4 ± 0.9	15 ± 1.2

All results represent mean ± standard deviation across 5-fold cross-validation with 3 independent runs.

**Table 7:** Ablation study.

Model	Accuracy (%)	F1-Score (%)	FPR (%)	Latency (ms)
CNN Only	91.8	91.2	7.9	12
BiLSTM Only	92.4	91.9	7.2	14
CNN + BiLSTM	93.7	93.1	6.1	15
CNN + BiLSTM + Multi-Metric	94.2	93.6	5.2	15
Full HM-MAD (Proposed)	**94.6**	**94.0**	**4.2**	15

## Data Availability

Data provided in the manuscript is available based on the request of corresponding author.

## Supplementary Materials

The following supporting information can be downloaded at https://www.mdpi.com/article/10.3390/s26123849/s1, Table S1: Computational overhead Metrics. Table S2: Statistical Significance Testing Results (Paired *t*-tests with Bonferroni Correction). Table S3: Real-World Performance Metrics—IoMT Deployment Scenarios.

## Abbreviations

Terms	Description/Typical Measurement
glucose_mgdl	Glucose level (mg/dL)
cgm_noise	Signal noise or variance
sensor_temp_c	Sensor temperature (°C)
pin_evt_gpio16	Pin event on GPIO16
reset_count	Device reset counter
boot_reason	Reason for last boot/reset
voltage_v	Voltage (V)
current_ma	Current draw (mA)
adc0_mv	ADC0 reading (mV)
spi_errors	SPI communication errors
uart_overruns	UART overrun errors
secure_boot	Secure boot enabled/flag
rssi_dbm	Received Signal Strength Indicator (dBm)
snr_db	Signal-to-Noise Ratio (dB)
packet_delay_ms	Packet delay/latency (ms)
jitter_ms	Variation in packet delay (ms)
packet_loss_pct	Packet loss percentage (%)
dup_rate_pct	Duplicate packet rate (%)
replay_flag	Replay attack indicator (bool/flag)
wifi_bssid	BSSID (MAC address) of AP
Channel	Wi-Fi channel
tx_power_dbm	Transmit power (dBm)
Auth	Authentication method/type
duty_cycle_pct	Device duty cycle(%)
UART Sniff	Universal Asynchronous Receiver-Transmitter Sniffing
MITM AP	Man-in-the-Middle Access Point
WBANs	Wireless Body Networks
IoMT	Internet of Medical Things
CNN	Convolution Neural Network
LSTM	Long Short-Term Memory
BiLSTM	Bidirectional Long Short-Term Memory
CGM	Continuous glucose monitoring
RSSI	Received Signal Strength Indicator
SVM	Support Vector Machine
FPR	False-Positive Rate
FNR	False-Negative Rate

## Appendix A

**Implementation Details and Hyperparameters**
**Hardware Platform**

The intrusion detection system was deployed on a **NodeMCU-32S (ESP32)** development board. The firmware was developed using **TensorFlow Lite for Microcontrollers (TFLite Micro)** and compiled with **Arduino IDE 2.3.2**. The model was exported from TensorFlow/Keras as a TFLite flatbuffer (.tflite) and loaded into flash memory. The compiled firmware occupied **580 kB (44%)** of program storage memory and **120 kB (37%)** of dynamic memory, leaving sufficient headroom for real-time operation.

**Dataset Characteristics**

The **cgm_wban_attack_dataset** contains **12,500 samples** across 12 classes (1 normal + 11 attack types), collected from a physical IoMT/WBAN testbed. Attacks were induced through the following:

- **Relay/timing**: software proxy adding 200 µs ± jitter delays;
- **Jamming**: Wi-Fi broadcast flood on same channel;
- **Replay**: packet duplication with sequence number replay;
- **Power glitch**: programmable PSU inducing brownouts (2.7V dips);
- **Firmware tamper**: unsigned firmware injection via OTA;
- **UART sniff**: debug traffic injection via GPIO/UART;
- **Sensor spoof**: CGM emulator with implausible glucose/temp values.

**Table A1:** Per-class distribution of training, validation, and testing samples in the IoMT/WBAN attack dataset.

Attack Type	Train	Val	Test	Total
Normal	2625	375	750	3750
Relay	1050	150	300	1500
Replay	840	120	240	1200
Jamming	735	105	210	1050
Timing	735	105	210	1050
Power Glitch	630	90	180	900
Firmware Tamper	630	90	180	900
UART Sniff	630	90	180	900
Battery Drain	630	90	180	900
Sensor Spoof	630	90	180	900
MITM AP	525	75	150	750
Total	8750	1250	2500	12,500

**Model Architecture**

The CNN-BiLSTM hybrid model processes input sequences of shape **(samples, n_features, 1)**.

**Table A2:** CNN-BiLSTM architecture details.

Layer	Type	Parameters	Output Shape	Notes
1	Conv1D	32 filters, kernel = 3, stride = 1, ReLU	(None, n_features-2, 32)	Same padding
2	MaxPooling1D	pool_size = 2	(None, ⌊(n_features-2)/2⌋, 32)	-
3	Dropout	rate = 0.3	Same	Regularization
4	BiLSTM	64 units (bidirectional)	(None, 128)	Concatenated forward+backward
5	Dense	64 units, ReLU	(None, 64)	-
6	Dropout	rate = 0.3	Same	Regularization
7	Dense	12 units, Softmax	(None, 12)	Final classification

**Total trainable parameters**: ~150 K (lightweight for NodeMCU).

**Training Protocol**

Models were trained on a **Google Colab GPU (T4)** using TensorFlow 2.15.

**Hyperparameters**:

- **Optimizer**: Adam (lr = 0.001, β_1_ = 0.9, β_2_ = 0.999;
- **Loss**: categorical cross-entropy;
- **Batch size**: 32;
- **Epochs**: 10 (with validation split);
- **Validation split**: 20% of training data;
- **Early stopping**: not used (fixed 10 epochs per script);
- **Callbacks**: none (can add ReduceLROnPlateau, EarlyStopping).

**Training runs**: **5 independent runs** with different random seeds (42, 123, 456, 789, 1011). All metrics in Table 6 are **mean ± standard deviation** over these 5 runs on the **held-out test set** (never seen during training/validation).

**Data preprocessing pipeline**:

Raw CSV → LabelEncoder (categorical→integer) → OneHotEncoder (integer→one-hot); → StandardScaler (Z-Score normalization) → Reshape (samples, n_features, 1).

### Rule-Based Hybrid Component

The rule-based module generates 4 binary flags:

- **timing_violation**: delay > 200 µs;
- **packet_drop**: packet_size == 0;
- **rssi_anomaly**: rssi < −90 or rssi > −20;
- **power_anomaly**: power < 1.8 V or power > 3.6 V.

Final decision: **ML prediction OR any rule flag → Alert + mitigation**.

**Table A3:** Hardware Performance Metrics.

Metric	Value	Notes
Flash usage	580 kB (44%)	Arduino IDE compile output
RAM usage	120 kB (37%)	Dynamic memory footprint
Inference latency	15 ± 1.2 ms	Mean over 1000 test samples
Idle current	70 mA @ 3.3V	USB power meter
Inference current	85 mA @ 3.3V	+21% during model execution

**Quantization**: Model was **8-bit integer quantized** post-training (TFLite converter) to reduce memory footprint by 75% while maintaining < 1% accuracy drop.
