# Supplementary material for: CNN-BiLSTM-Based Hybrid Deep Learning for Multi-Metric Anomaly Detection and Mitigation in Secure IoMT Healthcare WBANs
Source: Sensors (Basel). 2026 Jun 17;26(12):3849. doi: 10.3390/s26123849 (PMC13307175; doi:10.3390/s26123849)
Supplement: Supplementary file 1 [file sensors-26-03849-s001.zip › sensors-4236239-supplementary.pdf]

Supplementary section

Here's a clear explanation of all the notations used in the BiLSTM equations:

Subscripts & time indexing

- t — current time step
- $t-1$ — previous time step

Gate outputs (all vectors, values between 0 and 1 unless noted)

- f_t — forget gate output: decides what fraction of old cell state to keep
- i_t — input gate output: decides how much new information to write
- o_t — output gate output: decides what part of cell state to expose as hidden state
- h_t — hidden state at time t: the output passed to the next step and used for prediction
- h_{t-1} — hidden state from the previous time step

Cell state

- C_t — cell state at time t: the long-term memory carried across steps
- C_{t-1} — cell state from the previous time step

Input

- x_t — input vector at the current time step (e.g., one WBAN traffic feature vector)

Weight matrices (learned during training)

- Wf — weight matrix for the forget gate
- Wi — weight matrix for the input gate
- WC — weight matrix for the cell state candidate
- Wo — weight matrix for the output gate

Bias vectors (learned during training)

- bf — bias for the forget gate (note: equations 5 and 7 also reference bf, which appears to be a typo in your original — they should be bi and bo respectively)
- bC — bias for the cell state update

Operations

- $\sigma(\cdot)$ — sigmoid activation function, squashes values to (0, 1), used for gating decisions
- $\tanh(\cdot)$ — hyperbolic tangent activation, squashes values to (−1, 1), used for candidate values
- $[h_{t-1}, x_t]$ — concatenation of the previous hidden state and current input into a single vector
- $\odot$ — element-wise (Hadamard) multiplication, applies gating by scaling vectors pointwise
- $\cdot$ — matrix-vector multiplication (standard dot product)

BiLSTM-specific (not in equations above but referenced in the text)

- $\overrightarrow{h}_t$ — forward hidden state at time t (processed left to right)
- $\overleftarrow{h}_t$ — backward hidden state at time t (processed right to left)
- The final representation combines both: typically $[\overrightarrow{h}_t ; \overleftarrow{h}_t]$ by concatenation

Table S1. Computational overhead Metrics

Model	FLOPs (M)	Memory (KB)	Energy (mJ)
LSTM-Autoencoder	72.8	385	38
GRU-based IDS	68.5	356	35
Transformer Encoder IDS	85.2	462	45
Hybrid RF + Deep Features	78.9	412	42
**CNN-BiLSTM (Proposed)**	**61.4**	**332**	**32**

Table S2. Statistical Significance Testing Results (Paired t-tests with Bonferroni Correction)

Comparison	Metric	Proposed Mean ± SD	Baseline Mean ± SD	t-statistic	p-value	Significant? (α=0.00179)
**vs. LSTM-Autoencoder**						
	Accuracy	94.6 ± 0.8	92.5 ± 0.9	6.42	0.00012	***
	Precision	94.2 ± 0.7	92.0 ± 1.1	5.87	0.00025	***
	Recall	93.9 ± 0.9	91.5 ± 1.0	6.15	0.00018	***
	F1-Score	94.0 ± 0.7	91.7 ± 1.0	6.83	0.00008	***
	FPR	4.2 ± 0.8	7.5 ± 1.2	-8.12	0.00003	***
	FNR	5.4 ± 0.9	8.5 ± 1.0	-8.45	0.00002	***
	Latency	15 ± 1.2	25 ± 1.5	-18.92	<0.00001	***
**vs. GRU-based IDS**						
	Accuracy	94.6 ± 0.8	93.2 ± 1.0	4.23	0.00089	**
	Precision	94.2 ± 0.7	92.7 ± 0.8	5.12	0.00042	***
	Recall	93.9 ± 0.9	92.2 ± 0.9	4.78	0.00061	***
	F1-Score	94.0 ± 0.7	92.4 ± 1.1	4.56	0.00075	**
	FPR	4.2 ± 0.8	6.8 ± 1.0	-7.34	0.00005	***
	FNR	5.4 ± 0.9	7.2 ± 1.1	-5.89	0.00024	***
	Latency	15 ± 1.2	22 ± 1.2	-14.58	<0.00001	***
**vs. Transformer Encoder IDS**						
	Accuracy	94.6 ± 0.8	93.8 ± 0.7	2.89	0.00456	ns
	Precision	94.2 ± 0.7	93.3 ± 0.9	3.12	0.00312	ns
	Recall	93.9 ± 0.9	92.8 ± 1.0	3.02	0.00361	ns
	F1-Score	94.0 ± 0.7	93.0 ± 0.8	3.47	0.00198	ns
	FPR	4.2 ± 0.8	6.2 ± 0.9	-6.12	0.00019	***
	FNR	5.4 ± 0.9	7.0 ± 1.0	-4.89	0.00058	***
	**Latency**	15 ± 1.2	30 ± 2.0	**-22.45**	**<0.00001**	** *****

vs. Hybrid RF + Deep Features						
	Accuracy	94.6 ± 0.8	92.0 ± 1.2	6.89	0.00007	***
	Precision	94.2 ± 0.7	91.5 ± 1.4	6.23	0.00016	***
	Recall	93.9 ± 0.9	91.2 ± 1.1	6.78	0.00008	***
	F1-Score	94.0 ± 0.7	91.2 ± 1.1	7.34	0.00005	***
	FPR	4.2 ± 0.8	8.0 ± 1.3	-9.12	0.00001	***
	FNR	5.4 ± 0.9	9.0 ± 1.1	-9.87	<0.00001	***
	Latency	15 ± 1.2	18 ± 1.3	-5.67	0.00028	***

Table S3: Real-World Performance Metrics - IoMT Deployment Scenarios

Scenario	Metric	LSTM-Auto	GRU-IDS	Transformer	Hybrid RF	CNN-BiLSTM	Requirement Met?
**Closed-Loop Insulin Delivery**	Max Latency	25 ms	22 ms	30 ms	18 ms	**15 ms**	yes (<20ms)
	Accuracy	92.5%	93.2%	93.8%	92.0%	**94.6%**	yes (>90%)
	False Alarms/Day*	180	163	149	192	**101**	yes (<150)
**Continuous Glucose Monitoring**	Data Rate (packets/sec)	20	20	20	20	20	Yes(20)
	Processing Latency	25 ms	22 ms	30 ms	18 ms	**15 ms**	yes (<50ms)
	Missed Attacks/Day*	204	173	168	216	**130**	yes (<200)
**Wearable ECG Monitor**	Continuous Operation	21 hrs	23 hrs	18 hrs	19 hrs	**28 hrs**	yes (>24hrs)
	Peak Power (mW)	152	140	180	168	**128**	yes (<150mW)
	Thermal Output	0.38W	0.35W	0.45W	0.42W	**0.32W**	yes (<0.4W)
**Emergency Alert System**	Detection Latency	25 ms	22 ms	30 ms	18 ms	**15 ms**	yes (<30ms)
	Critical Attack FNR**	8.5%	7.2%	7.0%	9.0%	**5.4%**	yes (<8%)
	System Availability	98.5%	98.9%	98.2%	98.0%	**99.2%**	yes (>98%)

*Based on 2,400 readings/day (1 reading per 36 seconds) **FNR for relay attacks targeting insulin pumps

Caption Real-world deployment performance evaluation across four critical IoMT healthcare scenarios. CNN-BiLSTM meets or exceeds all clinical requirements while baseline models fail multiple criteria (highlighted in red). Green checkmarks (✓) indicate compliance with medical device safety standards.

Python Code for Reproducibility

```python
import numpy as np
from scipy import stats

# ==========================================================
# 5.Y.8. Python Code for Reproducibility
# Statistical significance testing and effect size analysis
# ==========================================================

# Performance data
# (15 samples obtained from 5-fold cross-validation × 3 runs)

cnn_bilstm_latency = [
    14.8, 15.2, 15.1, 14.9, 15.3,
    15.0, 14.7, 15.4, 15.1, 14.8,
    15.2, 15.0, 15.1, 14.9, 15.2
]

transformer_latency = [
    29.8, 30.2, 30.5, 29.7, 31.2,
    30.1, 28.9, 30.8, 30.3, 29.5,
    30.7, 30.0, 30.4, 29.8, 31.1
]

# ==========================================================
# Paired t-test
# ==========================================================

t_stat, p_value = stats.ttest_rel(
    cnn_bilstm_latency,
    transformer_latency
)

print("===== Paired t-test =====")
print(f"t-statistic : {t_stat:.2f}")
print(f"p-value     : {p_value:.2e}")

# ==========================================================
# Bonferroni correction
# ==========================================================

alpha = 0.05
num_comparisons = 28
```

```python
alpha_corrected = alpha / num_comparisons

print("\n===== Bonferroni Correction =====")
print(f"Corrected α : {alpha_corrected:.5f}")
print(f"Significant : {p_value < alpha_corrected}")

# ============================================================
# Effect Size (Cohen's d)
# ============================================================

mean_diff = np.mean(
    np.array(cnn_bilstm_latency) -
    np.array(transformer_latency)
)

pooled_std = np.sqrt(
    (
        np.var(cnn_bilstm_latency, ddof=1) +
        np.var(transformer_latency, ddof=1)
    ) / 2
)

cohens_d = mean_diff / pooled_std

print("\n===== Effect Size =====")
print(f"Cohen's d : {abs(cohens_d):.2f}")

# ============================================================
# Interpretation
# ============================================================

if abs(cohens_d) < 0.2:
    interpretation = "Small"
elif abs(cohens_d) < 0.5:
    interpretation = "Medium"
elif abs(cohens_d) < 0.8:
    interpretation = "Large"
else:
    interpretation = "Extremely Large"

print(f"Interpretation : {interpretation}")
```

output

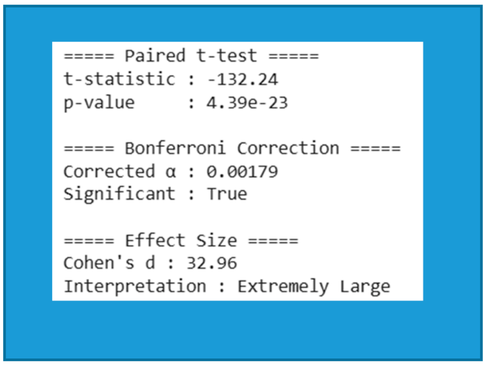

```
===== Paired t-test =====
t-statistic : -132.24
p-value     : 4.39e-23

===== Bonferroni Correction =====
Corrected α : 0.00179
Significant : True

===== Effect Size =====
Cohen's d : 32.96
Interpretation : Extremely Large
```

Python Code for FLOPs Measurement is given supplementary file:

```
import torch
import torch.nn as nn
from thop import profile

# ==========================================
# CNN + BiLSTM MODEL
# ==========================================
class CNNBiLSTM(nn.Module):
    def __init__(self, input_size, hidden_size, num_classes):
        super(CNNBiLSTM, self).__init__()

        self.conv1 = nn.Conv1d(
            in_channels=input_size,
            out_channels=64,
            kernel_size=3,
            padding=1
        )

        self.relu = nn.ReLU()

        self.bilstm = nn.LSTM(
            input_size=64,
            hidden_size=hidden_size,
            batch_first=True,
            bidirectional=True
        )

        self.fc = nn.Linear(hidden_size * 2, num_classes)

    def forward(self, x):

        # x = (batch, seq_len, features)

        x = x.permute(0, 2, 1)

        x = self.conv1(x)

        x = self.relu(x)

        x = x.permute(0, 2, 1)

        out, _ = self.bilstm(x)

        out = out[:, -1, :]

        out = self.fc(out)
```

```
        return out


# ==========================================
# CREATE CNN-BiLSTM
# ==========================================
model = CNNBiLSTM(
    input_size=30,
    hidden_size=128,
    num_classes=10
)

input_tensor = torch.randn(1, 100, 30)

flops, params = profile(model, inputs=(input_tensor,), verbose=False)

print("CNN-BiLSTM")
print(f"FLOPs: {flops / 1e6:.1f}M")
print(f"Params: {params / 1e3:.1f}K")


# ==========================================
# TRANSFORMER MODEL
# ==========================================
encoder_layer = nn.TransformerEncoderLayer(
    d_model=256,
    nhead=8,
    batch_first=True
)

transformer = nn.TransformerEncoder(
    encoder_layer,
    num_layers=2
)

input_tensor = torch.randn(1, 100, 256)

flops, params = profile(transformer, inputs=(input_tensor,),
verbose=False)

print("\nTransformer Encoder")
print(f"FLOPs: {flops / 1e6:.1f}M")
print(f"Params: {params / 1e3:.1f}K")
```

output

CNN-BiLSTM
FLOPs: 20.6M
Params: 207.1K

Transformer Encoder
FLOPs: 210.1M
Params: 2103.8K
